# Genetic Variation in Kruppel like Factor 15 Is Associated with Left Ventricular Hypertrophy in Patients with Type 2 Diabetes: Discovery and Replication Cohorts

**DOI:** 10.1016/j.ebiom.2017.03.036

**Published:** 2017-03-30

**Authors:** Sheila K. Patel, Bryan Wai, Chim C. Lang, Daniel Levin, Colin N.A. Palmer, Helen M. Parry, Elena Velkoska, Stephen B. Harrap, Piyush M. Srivastava, Louise M. Burrell

**Affiliations:** a Department of Medicine, University of Melbourne, Austin Health, Melbourne, Australia; b Department of Cardiology, Austin Health, Melbourne, Australia; c Division of Molecular and Clinical Medicine, Ninewells Hospital and Medical School, University of Dundee, Dundee, UK; d Pat McPherson Centre for Pharmacogenomics and Pharmacogenetics, Division of Molecular and Clinical Medicine, Ninewells Hospital and Medical School, University of Dundee, Dundee, UK; e Department of Physiology, University of Melbourne, Victoria, Australia

**Keywords:** Kruppel like factor 15, Left ventricular hypertrophy, Type 2 diabetes, Genetic association study, Echocardiogram, Heart failure

## Abstract

Left ventricular (LV) hypertrophy (LVH) is a heritable trait that is common in type 2 diabetes and is associated with the development of heart failure. The transcriptional factor Kruppel like factor 15 (KLF15) is expressed in the heart and acts as a repressor of cardiac hypertrophy in experimental models. This study investigated if *KLF15* gene variants were associated with LVH in type 2 diabetes. In stage 1 of a 2-stage approach, patients with type 2 diabetes and no known cardiac disease were prospectively recruited for a transthoracic echocardiographic assessment (Melbourne Diabetes Heart Cohort) (n = 318) and genotyping of two *KLF15* single nucleotide polymorphisms (SNPs) (rs9838915, rs6796325). In stage 2, the association of *KLF15* SNPs with LVH was investigated in the Genetics of Diabetes Audit and Research in Tayside Scotland (Go-DARTS) type 2 diabetes cohort (n = 5631). The *KLF15* SNP rs9838915 A allele was associated in a dominant manner with LV mass before (*P* = 0.003) and after (*P* = 0.001) adjustment for age, gender, body mass index (BMI) and hypertension, and with adjusted septal (*P* < 0.0001) and posterior (*P* = 0.004) wall thickness. LVH was present in 35% of patients. Over a median follow up of 5.6 years, there were 22 (7%) first heart failure hospitalizations. The adjusted risk of heart failure hospitalization was 5.5-fold greater in those with LVH and the rs9838915 A allele compared to those without LVH and the GG genotype (hazard ratio (HR) 5.5 (1.6–18.6), *P* = 0.006). The association of rs9838915 A allele with LVH was replicated in the Go-DARTS cohort. We have identified the *KLF15* SNP rs9838915 A allele as a marker of LVH in patients with type 2 diabetes, and replicated these findings in a large independent cohort. Studies are needed to characterize the functional importance of these results, and to determine if the SNP rs9838915 A allele is associated with LVH in other high risk patient cohorts.

## Highlights

• *KLF15* SNP rs9838915 A allele is associated with increased LV mass in patients with 2 diabetes.
• *KLF15* SNP rs9838915 predicts incident heart failure hospitalization.
• Genotyping *KLF15* SNP rs9838915 allowed more precise stratification of the risk of heart failure hospitalization.

Left ventricular hypertrophy (LVH) is a heritable trait that is common in patients with diabetes. The Kruppel like factor 15 (KLF15) is expressed in the heart and acts as a repressor of cardiac hypertrophy and fibrosis. Our study provides evidence that genetic variation in *KLF15* is associated with LVH in patients with type 2 diabetes and these findings were then replicated in an independent cohort of patients with type 2 diabetes. The *KLF15* genetic variant was also associated with first heart failure hospitalization. These findings add to our understanding of the molecular mechanisms that contribute to increased LV mass.

## 1 Introduction

Left ventricular (LV) hypertrophy (LVH) is a heritable trait associated with adverse cardiovascular outcomes including heart failure (Levy et al., 1990, de Simone et al., 2002) which is prevalent in type 2 diabetes (Shah et al., 2015). Although diabetes is an independent predictor of LVH, not all patients with diabetes develop LVH, suggesting that there is a genetic component to LV mass and the development of LVH. The genome wide association studies (GWAS) of LVH in the general population have identified a number of genetic loci associated with electrocardiographic or echocardiographic LVH, but these loci explain only a very small proportion of the variance (Vasan et al., 2009, Shah et al., 2011).

More precise phenotyping in populations at high risk of LVH such as patients with diabetes and a more detailed understanding of the molecular mechanisms that contribute to increased LV mass are needed. With regards to the latter, the Kruppel like factors (KLF) which are members of the zinc-finger class of DNA-binding transcriptional factors, have emerged as important regulators of cell growth and differentiation in experimental animal models (McConnell and Yang, 2010, Prosdocimo et al., 2015). To date, 18 family members have been identified including KLF15 which is highly expressed in cardiac myocytes and acts as a repressor of pathological cardiac hypertrophy (Wang et al., 2008, Fisch et al., 2007, Haldar et al., 2010). Cardiac KLF15 expression is induced postnatally and down regulated in response to pressure overload and hypertrophic stimuli (Fisch et al., 2007, Haldar et al., 2010). KLF15 null mice develop cardiac hypertrophy and heart failure in response to pressure overload, whilst overexpression of KLF15 reduces cell size (Fisch et al., 2007) and prevents the development of angiotensin II induced hypertrophy (Leenders et al., 2012). In addition to repressing cardiac hypertrophy, KLF15 is a transcriptional inhibitor of cardiac fibrosis (Wang et al., 2008). In neonatal rat ventricular fibroblasts, transforming growth factor-β1 (TGFβ1) reduced KLF15 expression and induced expression of connective tissue growth factor (CTGF), which is a key mediator of fibrosis (Candido et al., 2003); overexpression of KLF15 decreased both TGFβ1 and CTGF gene expression (Yu et al., 2015).

There is also evidence that loss of cardiac KLF15 expression may contribute to pathological LVH in man with downregulation of ventricular KLF15 expression in patients with LVH secondary to aortic stenosis (Fisch et al., 2007). In patients with end-stage heart failure undergoing a left ventricular assist implantation and explantation as a bridge to transplantation, KLF15 was reduced in the failing heart compared to control hearts, with significant recovery of KLF15 expression after mechanical unloading (Prosdocimo et al., 2014).

On the basis of the available experimental data, we hypothesised that *KLF15* *gene variants* are associated with LV mass in man. The *KLF15* gene is located on chromosome 3q21-q22 and comprises of 3 exons spanning over 14.8 kb of genomic DNA transcribed into a 416 amino acid protein. Two SNPs (rs9838915 G/A, rs6796325 T/C) capture all the common variation across the *KLF15* gene. As diabetes is associated with increased risk of LVH, we conducted our investigation in patients with type 2 diabetes. We used a 2-stage approach; Stage 1 examined the association between the 2 common *KLF15* haplotype tagging SNPs in Caucasian patients with type 2 diabetes and no known cardiac disease who were prospectively recruited for an echocardiographic assessment (Melbourne Diabetes Heart Cohort, Australia). We also determined if the *KLF15* SNPs predicted the adverse consequence of heart failure hospitalization. Stage 2 aimed to replicate the genetic findings in an independent cohort of Caucasian type 2 diabetes patients with an echocardiographic assessment of LVH who were recruited as part of the Genetics of Diabetes Audit and Research in Tayside (Go-DARTS) study (Parry et al., 2013).

## 2 Methods

A 2-stage approach was used to genotype the two common *KLF15* tagging SNPs. The Melbourne Diabetes Heart Cohort contributed to the discovery (stage 1) phase, and the Go-DARTS cohort contributed data to the replication (stage 2) phase. A description of the patient cohorts follows.

### 2.1 Stage 1 – Discovery Cohort

The Melbourne Diabetes Heart Cohort includes patients with type 2 diabetes and no known cardiac disease who were prospectively recruited at the time of transthoracic echocardiography, which was performed as part of the diabetes complications surveillance program at Austin Health, Melbourne (Srivastava et al., 2008, Patel et al., 2012a, Patel et al., 2012b). Patients with a clinically indicated echocardiogram, a past history of heart failure or with moderate/severe valvular dysfunction on the echocardiographic study were excluded. Patients of non-European ancestry were excluded. Ethical approval was obtained from the Human Research Ethics Committee at Austin Health, Melbourne and the study complied with the Declaration of Helsinki. All patients gave informed written consent. Height and weight and blood pressure were measured. Hypertension was defined as present if patients had a history of hypertension, were on anti-hypertensive medication and/or had evidence of hypertension (clinic blood pressure > 140/90 mm Hg) (American Diabetes Association, 2017). Glycosylated hemoglobin (HbA1c) and kidney function were measured and whole blood was collected in EDTA tubes and stored at − 80 °C for DNA extraction.

#### 2.1.1 Echocardiography

Transthoracic echocardiography was performed as previously described (Wai et al., 2014) according to the recommendations of the American Society of Echocardiography (ASE) (Lang et al., 2015). LV mass was calculated by LV cavity dimensions and wall thickness at end-diastole with the ASE recommended formula as follows (Lang et al., 2015): LV mass (g) = 0.8 × {1.04[(LVEDD + PWTd + SWTd)^3^ – (LVEDD)^3^]} + 0.6 g, where LVEDD is the LV end diastolic dimension, PWTd is the posterior wall thickness diameter and SWT is the septal wall thickness. Body surface area was calculated using the Mosteller formula (square root ([height (cm) × weight (kg)]/3600)) and LV mass was indexed to the body surface area. LVH was defined as LV mass index (LVMI) > 115 g/m^2^ in men and > 95 g/m^2^ in women (Lang et al., 2015).

#### 2.1.2 Genotyping

Genomic DNA was extracted as previously described (Patel et al., 2012b). Analysis of the linkage disequilibrium (LD) (r^2^) and *KLF15* haplotype structure was performed using the HapMap Phase II project data from the CEPH population and the Haploview software (version 4·2) (Barrett et al., 2005). *KLF15* haplotype tagging SNPs were identified using pairwise r^2^ thresholds of 0.8 and minor allele frequency of 0.05. Two *KLF15* tagging SNPs, rs9838915 G/A and rs6796325 T/C captured all the common variation across the *KLF15* gene and were genotyped using the Sequenom MassARRAY system (Sequenom, San Diego, CA, USA). Genotyping included 10% duplicate samples and negative controls per 96 well plate. Genotyping concordance between duplicate samples was 100% for both SNPs.

#### 2.1.3 Clinical Endpoint - Heart Failure Hospitalization

We also assessed incident heart failure hospitalization in the discovery cohort. Clinical outcomes were obtained via medical records review with all patients having ongoing follow-up at our institution. The Framingham criteria were used for the clinical diagnosis of heart failure (McKee et al., 1971). Two major criteria or 1 major and 2 minor criteria which occurred concurrently were required for the diagnosis; major criteria included symptoms such as paroxysmal nocturnal dyspnea or/and orthopnea, clinical features such as elevated jugular venous pressure, presence of pulmonary rales and/or presence of a third heart sound and radiological evidence with presence of cardiomegaly and/or pulmonary oedema on chest x-ray. Minor criteria included the presence of peripheral oedema, night cough, dyspnea on exertion, hepatomegaly, pleural effusion, heart rate > 120 beats per minute and/or weight loss of > 4.5 kg in 5 days (considered as major criterion if it occurred during therapeutic intervention for heart failure) (Senni et al., 1998).

### 2.2 Stage 2 – Replication Cohort

The Diabetes Audit and Research in Tayside (DARTS) cohort has been described previously (Morris et al., 1997). Briefly, demographic, prescribing, echocardiographic, morbidity, mortality and genotyping databases connected with the Go-DARTS, Scotland project (Parry et al., 2013) were linked using a patient-specific identifier. The DARTS and Go-DARTS studies received ethical approval from the local boards. There were 5631 patients with type 2 diabetes recruited to Go-DARTS between December 1998 and May 2009.

#### 2.2.1 Echocardiography

The Tayside echocardiography database was used to identify echocardiographically defined LVH cases according to ASE criteria (Lang et al., 2015). This database contains information on all clinically requested echocardiograms performed at Ninewells Hospital. Individuals were classed as having LVH as previously described (Parry et al., 2013). Type 2 diabetes patients with aortic stenosis greater than mild severity were excluded. The Control subjects have been previously described (Parry et al., 2013). Briefly, control subjects had a diagnosis of type 2 diabetes, no clinically requested echocardiogram and had never received a prescription for a loop diuretic. Genetic samples from those meeting the inclusion criteria were taken forward for further analysis as non-LVH controls compared to LVH cases.

#### 2.2.2 Genotyping

Genotyping data from the Go-DARTS 1560 cases of LVH and 4071 non-LVH controls were analysed. Genotyping of samples and quality control have been previously described (Parry et al., 2013).

## 3 Statistical Analyses

### 3.1 Discovery Cohort

The analyses were performed using SPSS version 20 (IBM SPSS Statistics, IBM Corp, USA). The genotype frequencies were assessed for Hardy-Weinberg equilibrium using the Chi-square (χ^2^) test. To compare characteristics of patients by LVH status, the independent samples *t*-test was used for continuous variables, the Mann-Whitney *U* test for variables that were not normally distributed and the Pearson's χ^2^ test to compare categorical variables. Continuous variables that were normally distributed are presented as mean ± standard deviation (SD) and non-parametric variables are presented as medians [25th, 75th quartiles]. Single SNP effects with cardiac structure and function parameters were analysed using the dominant genetic model (AA vs. Aa/aa) using the independent samples *t*-test, where ‘A’ represents the major allele and ‘a’ represents the minor allele. The association of *KLF15 SNP* genotypes with echocardiographic cardiac parameters were examined further using multiple linear regression analysis after adjusting for known risk factors for increased LV mass (age, gender, body mass index (BMI) and hypertension) in the dominant genetic model.

Patients were stratified into 4 groups according to the presence of LVH and the SNP rs9838915 genotype as follows: LVH and rs9838915 A allele carriers (GA/AA), LVH and rs9838915 GG homozygotes, no LVH and rs9838915 A allele carriers and no LVH and rs9838915 GG homozygotes. Unadjusted Kaplan-Meier analysis was performed to estimate the cumulative percentage of first heart failure hospitalization and compared using the log-rank test statistic according to the presence or not of LVH, and then according to the presence or not of LVH stratified by the SNP rs9838915 genotype. Cox regression was used to analyse the association between LVH and heart failure hospitalization, and also with LVH stratified by SNP rs9838915 genotype. Cox regression analysis included significant clinical variables (*P* < 0.05) from Table 1 and BMI and hypertension, which are known risk factors for the development of heart failure. Patients without LVH and rs9838915 GG homozygotes were used as the reference group. The risk of heart failure hospitalization between patients with LVH and GA/AA genotype and patients with LVH who were GG homozygotes was also compared. The results are reported as hazard ratio (HR) and 95% confidence intervals (CI). Two-tailed *P*-values < 0.05 were considered significant.

### 3.2 Replication Cohort

Linear regression analysis was carried out in the Go-DARTS cohort for the *KLF15* SNP with LVH. The analyses were performed using R version 3.1.0. Characteristics of patients by LVH status were compared as for the discovery cohort. Association of alleles with LVH was tested for each SNP using Chi-squared tests. The association of *KLF15 SNP* genotypes with cardiac parameters were examined further using multiple logistic regression analysis after adjusting for known risk factors for increased LV mass (age, gender, BMI, systolic and diastolic blood pressure) in the rs9838915 AA allele compared to the GG reference genotype or the dominant genetic model (GG vs. GA/AA). The results are reported as odds ratio and 95% CI. Two-tailed *P*-values < 0.05 were considered significant.

## 4 Results

### 4.1 KLF15 SNPs rs9838915 and rs6796325

**Fig. 1:**
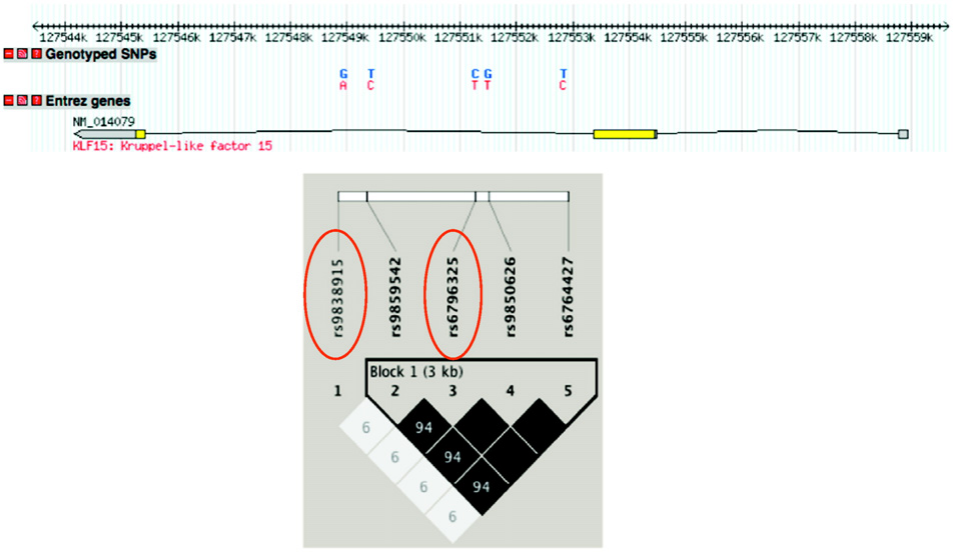
Linkage disequilibrium structure at the *KLF15* gene. Haploview image of the pairwise linkage disequilibrium (r^2^) between SNPs with minor allele frequencies > 5% based on the HapMap genotypes from the CEU population. There is one haplotype block. The pairwise r^2^ is indicated in the square boxes, blank squares represent r^2^ values of 100. The darker the squares the higher the pairwise r^2^ between the SNPs. The tag SNPs are circled in red.

The two *KLF15* tagging SNPs (rs9838915, rs6796325) captured all the common variation (minor allele frequencies > 5%) in the *KLF15* gene region from HapMapII (captured 20 SNPs with an r^2^ > 0·8 and mean r^2^ of 0.99, Fig. 1). Genotype frequencies are shown in Table 1 for the 2 cohorts. In the discovery cohort, the frequency of the *KLF15* rs9838915 G and A alleles were 85% and 15% respectively and the *KLF15* rs6796325 T and C alleles were 71% and 29% respectively. In the replication cohort, the frequency of the *KLF15* rs9838915 G and A alleles were 82% and 18% respectively and the *KLF15* rs6796325 T and C alleles were 77% and 23% respectively. These allele frequencies were similar to the allele frequency of the CEPH (Utah residents with ancestry from northern and western Europe) studied in the HapMap project (International HapMap Consortium, 2003). The distribution of genotypes was in Hardy-Weinberg equilibrium (*P* > 0.05).

### 4.2 Stage 1 – Discovery Cohort

Table 2 shows the clinical characteristics of the discovery cohort which included 318 patients (172 men, 146 women) aged 63.9 ± 11.7 years (mean ± SD) with a BMI of 31.7 ± 6.1 kg/m^2^ and median [25th, 75th quartiles] diabetes duration of 10 [5, 16] years. Hypertension was present in 79% and oral hypoglycemic agents and/or insulin were used by > 90% of patients. LVH was present in 35% of patients. On univariate analysis, patients with LVH were older (*P* < 0.0001), more likely to be females (*P* < 0.0001) and had increased systolic blood pressure (*P* = 0.03). Kidney function and glycemic control did not differ between groups. Diabetes duration was similar in both groups (*P* = 0.05), and there was no difference in the number of patients on glucose lowering therapy. The echocardiographic characteristics are shown in Table 3. By definition those with LVH had increased LVMI and increased PWT, SWT and LVDD (all, *P* < 0.0001) compared to those without LVH. There were no differences in systolic function (ejection fraction) or diastolic function (E/e′) according to the presence or not of LVH.

#### 4.2.1 Association of KLF15 SNPs with Echocardiographic Variables

LVMI was significantly associated with *KLF15* SNP rs9838915 G/A in a model consistent with a dominant effect of the A allele (Table 4). After adjustment for known covariates of increased LV mass including age, gender, BMI and hypertension, the association of the A allele and LVMI was significant in the dominant genetic model (*P* = 0.001). In addition, the *KLF15* rs9838915 A allele was significantly associated with increased SWT in the adjusted dominant model (*P* < 0.0001) and with increased PWT (*P* = 0.004). More patients with the rs9838915 A allele had LVH (33% vs. 22·4%, *P* = 0.045) and the odds of having LVH with the A allele were 60% higher (odds ratio 1.6 (1.0–2.8), *P* = 0.046, data not shown). There were no significant SNP rs9838915 genotype associations with LV dimensions or ejection fraction. The *KLF15* rs6796325 T/C SNP was not associated with any echocardiographic parameter.

#### 4.2.2 Heart Failure Hospitalization and Stratification of LVH by KLF15 SNP rs9838915 Genotype

**Fig. 2:**
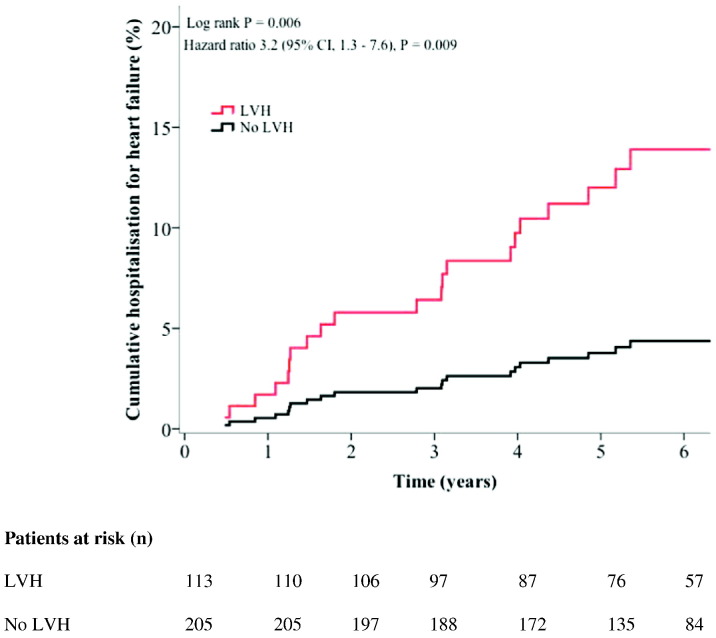
Discovery cohort: Unadjusted Cox regression analysis curve for the cumulative incidence of heart failure hospitalization according to the presence of left ventricular hypertrophy (LVH). Graph shows the Log rank *P* value from Kaplan-Meier analysis and the Cox regression analyses hazard ratio and *P* value.

Twenty-two patients were hospitalized with new onset heart failure over a median follow up time of 5.6 years (min – max 0.5 to 8.9 years). Kaplan-Meier analysis showed a significant difference in time to heart failure hospitalization by the presence or absence of LVH (Log rank *P* = 0.006). Fig. 2 shows the unadjusted Cox regression curve according to the presence or not of LVH; LVH was associated with a 3.2-fold risk in heart failure hospitalization (HR 3.2 (95% CI 1.3–7.6), *P* = 0.009). In multivariable Cox regression analysis (Table 5A), the presence of LVH remained significantly associated with a 3-fold increase in heart failure hospitalization (HR 3.0 (1.1–7·9) *P* = 0.029) independent of age, gender, BMI, systolic blood pressure and hypertension. Older age and BMI were also significant predictors of heart failure hospitalization.

**Fig. 3:**
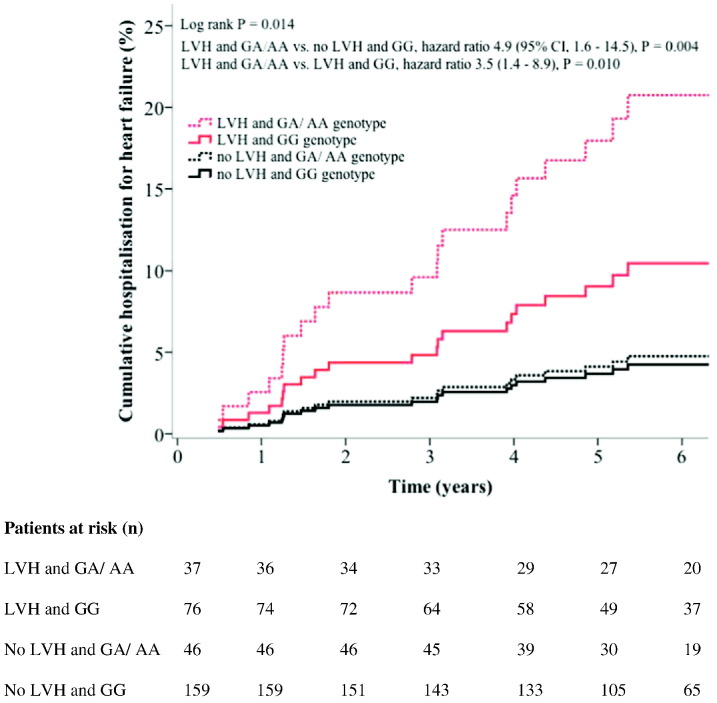
Discovery cohort: Unadjusted Cox regression analysis curve for the cumulative incidence of heart failure hospitalization stratified according to the presence or absence of left ventricular hypertrophy (LVH) and *KLF15* rs9838915 genotype. Graph shows the Log rank *P* value from Kaplan-Meier analysis and the Cox regression analyses hazard ratio and *P* value.

Fig. 3 shows the unadjusted Cox regression curve in patients according to the presence or not of LVH stratified according to the *KLF15* rs9838915 genotype (GG homozygotes or A allele carriers). Kaplan-Meier analysis showed a significant difference in time to heart failure hospitalization by the presence or not of LVH stratified by rs9838915 genotype (Log rank *P* = 0.014). The unadjusted risk of first heart failure hospitalization was nearly 5-fold greater in patients with LVH and the rs9838915 A allele, compared to those with no LVH and the GG genotype (HR 4.9 (1.6–14.5), *P* = 0.004) and 3.5 fold higher compared to those with LVH and the GG genotype (HR 3.5 (1.4–8.9), *P* = 0.010). In the adjusted Cox regression analysis (Table 5B) patients with LVH and A allele remained at increased risk of heart failure hospitalization (HR 5.5 (1.6–18.6), *P* = 0.006) compared to patients with no LVH/GG genotype, and were at a 3-fold higher risk then those with LVH/GG genotype (HR 3.3 (1.2–9.2), *P* = 0.020). There was a 10% increase in heart failure hospitalization risk with each year of increasing age (HR 1.1 (1.0–1.1), *P* = 0.005) and a 10% increase with each unit increase in BMI (HR 1.1 (1.0–1.2), *P* = 0.004).

### 4.3 Stage 2 – Replication Cohort

Table 2 shows the clinical characteristics of the replication cohort. This included 5631 patients (45% female) aged 65.5 ± 11.0 years (mean ± SD) with a BMI of 31.0 ± 5.7 kg/m^2^ and diabetes duration of 5.2 [2.7, 9.6] years. Oral hypoglycemic agents and/or insulin were used by 87% of patients. LVH was present in 28% of patients. On univariate analysis, patients with LVH were older (*P* < 0.0001), had longer diabetes duration (*P* = 0.002), and poorer kidney function (*P* < 0.0001). Glycemic control and BMI did not differ significantly between groups.

#### 4.3.1 Association of KLF15 SNPs with Echocardiographic LVH

Genotype frequencies are shown in Table 6A according to LVH status. Chi-squared tests for the presence of LVH showed significant association by genotype for the rs9838915 SNP (*P* = 0.049) and re-categorizing to with/without AA allele (with highest LVH prevalence), showed similar significance (*P* = 0.048). A multivariable logistic model of LVH presence including rs9838915 allele and relevant covariates (Table 6B) showed a significant effect for the AA allele compared to GG reference (odds ratio = 1.47, 95% CI = (1.05–2.06), *P* = 0.023). *Re*-categorizing by the dominant model (GG vs. GA/AA) gave an odds ratio (95% CI) of 1.09 (0.96–1.24), *P* = 0.17 for GA/AA vs. GG. An equivalent model for *KLF15* SNP rs6796325 showed no significance.

## 5 Discussion

This clinical study investigated the association between the *KLF15* gene and LVH in patients with type 2 diabetes. A key finding in the discovery cohort was that the A allele at rs9838915 SNP in the *KLF15* gene was associated with increased LV mass in patients with type 2 diabetes. Carriers of the A allele had a 14 g/m^2^ increase in LV mass compared to those with the GG genotype that was independent of age, gender, BMI and hypertension. Furthermore, we replicated the association of the *KLF15* SNP rs9838915 A allele with LVH in a large, independent cohort of patients with type 2 diabetes, the Go-DARTS cohort (*n* = 5631). We found no association of the *KLF15* rs6796325 T/C SNP with any echocardiographic parameter in either the discovery or the replication cohorts.

In a preliminary analysis, we explored the association between LVH, *KLF15* genotype and heart failure outcomes in the discovery cohort. Echocardiographic LVH was present in 35% of patients and was associated with a 3-fold increased risk of first heart failure hospitalization that was independent of age, gender, systolic blood pressure, BMI and hypertension. The adjusted risk of heart failure hospitalization was 5.5-fold greater in those with LVH and the *KLF15* rs9838915 A allele compared to the group with no LVH and the GG genotype. These results, albeit in a small cohort, suggest that patients with type 2 diabetes and LVH may be able to be more precisely stratified for risk of the development of heart failure according to their *KLF15* rs9838915 genotype.

Diabetes is an independent predictor of LVH, but not all patients with diabetes develop LVH, suggesting that there is a genetic component to LV mass and the development of LVH. In this study, patients had relatively well controlled diabetes (HbA_1c_ ~ 7–7.5%) with ~ 90% on glucose lowering therapy, but despite this a significant number had LVH. The genetic basis of LVH has been mainly studied in the general population, whereas we specifically chose to study patients at high risk of LVH due to the presence of diabetes. GWAS has identified some genetic loci associated with electrocardiographic or echocardiographic LVH (van der Harst et al., 2016) but none of the SNPs reached genome wide significance on chromosome 3, which is the location of the *KLF15* gene (Vasan et al., 2009). The EchoGen study (*n* = 12,612) investigated associations with echocardiographic LV mass in 5 community-based cohorts of European ancestry (Vasan et al., 2009) and Shah et al. investigated associations with ECG-LVH in 10,258 individuals in 3 population based cohorts (Shah et al., 2011). Neither study was enriched for risk factors for LVH such as hypertension, obesity and diabetes. A recent meta-analysis of GWAS in a much larger cohort of 73,518 European ancestry individuals identified 52 genomic loci associating with 4 ECG measured QRS complex phenotypes (van der Harst et al., 2016).

It is unknown how rs9838915 SNP located in intron 2 influences LV mass. KLF15 is expressed in cardiomyocytes and acts as a repressor of pathological cardiac hypertrophy (Wang et al., 2008, Fisch et al., 2007, Haldar et al., 2010, Leenders et al., 2012) through inhibition of the cardiac transcriptional factors GATA4 and MEF2 (Fisch et al., 2007). One possibility is that genetic variation disrupts the ability of KLF15 to repress hypertrophic transcriptional factors, leading to increased expression of these genes and thus cardiac hypertrophy. KLF15 is also a transcriptional inhibitor of cardiac fibrosis (Wang et al., 2008), which is a key feature of diabetic heart disease (Rubler et al., 1972). It is also possible that the observed genetic variation in *KLF15* could lead to increased cardiac fibrosis which would in turn contribute to increased LV mass and myocardial stiffness. Further studies are required to determine whether the association we observe is due to SNP rs9838915 or a neighboring SNP that may be in strong linkage disequilibrium with rs9838915. We performed a bioinformatics analysis to identify putative causal variants underlying the association of the *KLF15* SNP rs9838915 with LVH. We explored the co-localisation of the association signal with features indicative of functional genomic elements, including evidence of transcription factor binding, DNase hypersensitivity and histone modification marks. An analysis of the RegulomeDB suggests that SNP rs9838915 is located in a transcription factor binding site and an enhancer element. These functional elements were identified in human LV tissue. We used the SNAP tool (http://www.broadinstitute.org/mpg/snap) by the Broad Institute to identify SNPs that were in LD (r^2^ > 0.8) with SNP rs9838915. We identified a SNP located 635 bp upstream of rs9838915 that was highly correlated (r^2^ = 1.0) and located within an enhancer element identified in human LV, right atrium and ventricle tissue.

## 6 Strengths and Limitations

We conducted a clinical study investigating the association of *KLF15* with echocardiographically determined LV mass in patients at high risk of LVH due to type 2 diabetes. Our finding in the discovery cohort that the rs9838915 SNP A allele in the *KLF15* gene was associated with increased LV mass in type 2 diabetes was replicated in a large, independent cohort. Several limitations deserve comment. The results are restricted to patients with type 2 diabetes of Caucasian ethnicity, and future studies should examine patients without diabetes as well as patients of different ethnic backgrounds. Functional studies were not performed but are required to determine the exact mechanisms by which genetic variation in *KLF15* influences LV mass.

## 7 Conclusions

We identified a gene variant, the rs9838915 SNP in the *KLF15* gene that is relevant to increased LV mass in 318 patients with type 2 diabetes, and validated these findings in a large independent cohort of > 5000 individuals with type 2 diabetes. Studies are now needed to characterize the functional importance of these results, to understand the biological mechanisms involved, and to determine if the *KLF15* SNP rs9838915 A allele is associated with LVH in patients without diabetes.

## Funding

The work in the Melbourne Diabetes Heart Cohort was supported by a Diabetes Australia Research Program grant [grant number Y12G-PATS] to [S.K.P]; a Career Development Award, University of Melbourne [S.K.P]; National Heart Foundation of Australia [grant number G12M6368] to [L.M.B]; National Health and Medical Research Council of Australia/National Heart Foundation scholarship to [B.W]. The Go-DARTS study was supported by the following: genotyping was facilitated by capital funding from the Scottish Government Chief Scientist Office Generation Scotland initiative (www.generationscotland.org); The Wellcome Trust U.K. type 2 diabetes case control collection (GoDARTS2) was funded by a Wellcome Trust [grant number GR02960] and the GWAS genotyping was performed as part of the Wellcome Trust Case Control Consortium 2 [084726/Z/08/Z, 085475/Z/08/Z, 085475/B/08/Z]. Our funders had no role in the study design, data collection, data analysis, interpretation or in writing the manuscript.

## Conflicts of Interest

SBH reports personal fees from Servier, outside of the submitted work; LMB reports personal fees from Novartis and from AstraZenca, outside of the submitted work. All other authors have nothing to disclose.

## Author Contributions

SKP, CCL and LMB conceived the study, obtained the funding to conduct it, and wrote the manuscript. SKP, BW, CCL, PMS, LMB designed the study. SKP, CCL, DL, CNAP analysed the data. SKP, BW, CCL, DL, CNAP, EV, SBH, PMS, LMB interpreted the data. BW, DL, CNAP, HMP, EV, SBH, PMS commented and revised the manuscript.

## Figures and Tables

**Table 1:** Discovery and replication cohorts: descriptive information on *KLF15* SNPs and genotype frequencies.

SNPs^a^	Chromosome position^b^	Gene region position	Major/minor alleles	Discovery cohort		Replication cohort					
				Genotype frequencies, % (n)	MAF	Genotype frequencies, % (n)					MAF
rs9838915	1.26E + 08	Intron 2	G/A	GG	GA	AA	15	GG	GA	AA	18
				74.0 (235)	22.6 (72)	3.4 (11)		66.3 (3673)	30.5 (1691)	3.1 (174)	
rs6796325	1.26E + 08	Intron 2	T/C	TT	TC	CC	29	TT	TC	CC	23
				52.0 (165)	38.6 (123)	9.4 (30)		59.8 (3338)	35.1 (1959)	5.1 (285)	

MAF = minor allele frequency.

a dbSNP rs identification numbers (http://www.ncbi.nlm.nih.gov/SNP/, last accessed 2nd August 2016).

b Chromosome 3 SNP positions using the genomic contig NT_005612·17 (http://www.ncbi.nlm.nih.gov/nuccore/NT_005612.17, last accessed 2nd August 2016).

**Table 2:** Discovery and replication cohorts: clinical characteristics according to the absence or presence of LVH.

Characteristic	Discovery cohort				Replication cohort			
	All patients	No LVH	LVH	*P* value	All patients	No LVH	LVH	*P* value
n	318	205	113		5631	4071	1560	
Age (years)	63.9 ± 11.7	61.9 ± 12.0	67.7 ± 10·4	< 0.0001	65.5 ± 11·0	64.7 ± 11.4	67.6 ± 9.6	< 0.0001
Male gender, n (%)	172 (54)	127 (62)	45 (40)	< 0.0001	3106 (55.2)	2268 (55.7)	838 (53.7)	0.19
Diabetes duration (year)^a^	10 [5, 16]	10 [5, 16]	12 [6, 18]	0.05	5.2 [2.7, 9.6]	5.1 [2.6, 9.4]	5.6 [2.8, 10.2]	0.002
BMI (kg/m^2^)	31.7 ± 6.1	31.7 ± 5.8	31.7 ± 6.5	0.99	31.0 ± 5.7	30.9 ± 5.7	31.0 ± 5.4	0.56
HbA_1c_ (%)	7.7 ± 1.3	7.6 ± 1.3	7.9 ± 1.2	0.06	7.4 ± 1.1	7.4 ± 1.1	7.4 ± 1.1	0.33
eGFR (ml/min/1.73m^2^)	71 ± 21	73 ± 20	68 ± 22	0.08	71% ≥ 60	77% ≥ 60	55% ≥ 60	< 0.0001
Systolic blood pressure (mm Hg)	138 ± 20	136 ± 19	141 ± 21	0.03	140 ± 11	140 ± 10	141 ± 12	0.0055
Diastolic blood pressure (mm Hg)	76 ± 10	76 ± 10	75 ± 9	0.36	78 ± 6.3	78 ± 6.1	77 ± 6.6	< 0.0001
Hypertension, n (%)	250 (79)	157 (77)	93 (82)	0.23	–	–	–	–
Oral hypoglycaemics and/or insulin, n (%)	288 (91)	189 (92)	99 (88)	0·18	4893 (86.9)	3537 (86.9)	1356 (86.9)	1.00

Data is expressed as mean ± SD.

a Median [25th, 75th quartiles] or n (%). BMI, body mass index; eGFR, estimated glomerular filtration rate.

**Table 3:** Discovery cohort: echocardiographic characteristics according to the absence or presence of LVH.

Characteristic	All patients	No LVH	LVH	*P* value
n	318	205	113	
Echocardiographic parameters				
*Cardiac structure*				
LVMI (g/m^2^)	98.5 ± 26.6	84.6 ± 18.9	123.8 ± 18.7	< 0.0001
LVH, n (%)	113 (35)	0	113 (100)	
Posterior wall thickness (cm)	1.05 ± 0.15	1.00 ± 0.15	1.11 ± 0.14	< 0.0001
Septal wall thickness (cm)	1.07 ± 0.15	1.03 ± 0.15	1.15 ± 0.14	< 0.0001
LVEDD (cm)	4.9 ± 0.7	4.7 ± 0.7	5.3 ± 0.6	< 0.0001
LVESD (cm)	3.1 ± 0.8	3.1 ± 0.8	3.2 ± 0.7	0.19
*Cardiac function*				
Ejection fraction (%)	68 ± 12	69 ± 10	68 ± 13	0.38
E (m/s)	0.82 ± 0.20	0.82 ± 0.19	0.82 ± 0.23	0.88
A (m/s)	0.89 ± 0.22	0.88 ± 0.22	0.91 ± 0.23	0.37
E/A ratio	0.95 ± 0.29	0.97 ± 0.30	0.90 ± 0.28	0.06
Deceleration time (ms)	229.4 ± 60.7	226.6 ± 57.5	234.2 ± 66.3	0.30
e′ (m/s)	0.09 ± 0.04	0.09 ± 0.04	0.08 ± 0.04	0.20
E/e′ ratio^a^	10.1 [7.2, 14.0]	9.9 [7.1, 13.7]	10.7 [7.6, 14.1]	0.42

Data is expressed as mean ± SD.

a Median [25th, 75th quartiles] or n (%). LVH, left ventricular hypertrophy; LVEDD, left ventricular end diastolic dimension; LVESD, left ventricular end systolic dimension; LVMI, left ventricular mass index.

**Table 4:** Discovery cohort: relationship between *KLF15 SNP* genotypes and echocardiographic parameters.

	Genotypes			*P* values for dominant model	
				Unadjusted	Adjusted^a^
*KLF15* rs9838915	GG	GA	AA		
n	235	72	11		
LVMI (g/m^2^)	95.9 ± 25.5	105.3 ± 28.8	109.3 ± 25.9	0.003	0.001
Posterior wall thickness (cm)	1.04 ± 0.15	1.08 ± 0.15	1.07 ± 0.13	0.032	0.004
Septal wall thickness (cm)	1.05 ± 0.15	1.11 ± 0.14	1.08 ± 0.16	0.003	< 0.0001
LVEDD (cm)	4.9 ± 0.7	4.9 ± 0.7	5.1 ± 0.5	0.69	0.96
LVESD (cm)	3.1 ± 0.8	3.2 ± 0.8	3.1 ± 0.4	0.96	0.11
Ejection fraction (%)	68 ± 11	68 ± 12	69 ± 9	0.59	0.28

*KLF15* rs6796325	TT	TC	CC		
n	165	123	30		
LVMI (g/m^2^)	97.6 ± 25.6	101.3 ± 27.8	95.0 ± 27.0	0.34	0.69
Posterior wall thickness (cm)	1.04 ± 0.14	1.06 ± 0.16	1.03 ± 0.15	0.69	0.34
Septal wall thickness (cm)	1.06 ± 0.15	1.08 ± 0.16	1.06 ± 0.13	0.52	0.60
LVEDD (cm)	4.9 ± 0.8	4.9 ± 0.7	4.9 ± 0.9	0.31	0.27
LVESD (cm)	3.1 ± 0.8	3.1 ± 0.8	3.4 ± 0.8	0.40	0.44
Ejection fraction (%)	68 ± 11	69 ± 12	66 ± 16	0.83	0.28

Data is expressed as mean ± standard deviation.

a Adjusted for known risk factors for increased LV mass (age, gender, BMI, hypertension). LVEDD, left ventricular end diastolic dimension; LVESD, left ventricular end systolic dimension; LVMI, left ventricular mass index.

**Table 5:** Discovery cohort.

A. Cox regression analysis of independent predictors of incident heart failure hospitalization according to the presence of LVH adjusted for known predictors of heart failure		
Variables	HR (95% CI)	*P* value
Presence of LVH	3.0 (1.1–7.9)	0.029
Age (years)	1.1 (1.0–1.1)	0.006
Male gender	2.5 (0.9–7.2)	0.084
BMI (kg/m^2^)	1.1 (1.0–1.2)	0.003
Systolic blood pressure (mmHg)	1.0 (0.9–1.0)	0.504
Hypertension	2.4 (0.3–21.2)	0.416


B: Cox regression analysis of independent predictors of heart failure according to the presence of LVH stratified by *KLF15* rs9838915 genotype adjusted for known predictors of heart failure		
Variables	HR (95% CI)	*P* value
No LVH and GA/AA genotype^a^	1.9 (0.4–9.8)	0.462
LVH and GG genotype^a^	2.4 (0.7–8.4)	0.165
LVH and GA/AA genotype^a^	5.5 (1.6–18.6)	0.006
LVH and GA/AA genotype^b^	3.3 (1.2–9.2)	0.020
Age (years)	1.1 (1.0–1.1)	0.005
Male gender	2.4 (0.9–6.9)	0.098
BMI (kg/m^2^)	1.1 (1.0–1.2)	0.004
Systolic blood pressure (mm Hg)	1.0 (0.9–1.0)	0.458
Hypertension	3.2 (0.3–30.6)	0.313

LVH, left ventricular hypertrophy; BMI, body mass index.

a Hazard ratio compared to no LVH and GG genotype.

b Hazard ratio compared to patients with LVH and GG genotype.

**Table 6:** Replication cohort.

A. Genotype distribution by LVH					
KLF15 SNP	Allele	Case	Control	Total	*P* value
rs9838915	GG	985 (26.8%)	2688 (73.2%)	3673	0.049
	GA	483 (28.6%)	1208 (71.4%)	1691	
	AA	60 (34.5%)	114 (65.5%)	174	
	Total	1528 (27.6%)	4010 (72.4%)	5538	
rs6796325	TT	939 (28.1%)	2399 (71.9%)	3338	0.54
	TC	537 (27.4%)	1422 (72.6%)	1959	
	CC	72 (25.3%)	213 (74.7%)	285	
	Total	1548 (27.7%)	4034 (72.3%)	5582	


B: Logistic regression analyses for LVH with SNP rs9838915			
	Estimate	Std. Error	*P* value
rs9838915 GA	0.06783	0.06824	0.32
rs9838915 AA	0.38727	0.17054	0.023
Male gender	0.17931	0.06840	0.008
Age (years)	0.01095	0.00373	0.003
BMI (kg/m^2^)	0.02742	0.00612	< 0.0001
Systolic blood pressure (mm Hg)	− 0·14612	0.02785	< 0.0001
Diastolic blood pressure (mmHg)	− 0.33324	0.05179	< 0.0001
